# Prevalence of Exocrine Pancreatic Insufficiency in Women with Obesity Syndrome: Assessment by Pancreatic Fecal Elastase 1

**DOI:** 10.5402/2011/951686

**Published:** 2011-11-03

**Authors:** J. Teichmann, J. F. Riemann, U. Lange

**Affiliations:** ^1^Department of Gastroenterology, Endocrinology, and Diabetology, Klinikum Lüdenscheid, 58515 Lüdenscheid, Germany; ^2^Department of Internal Medicine, Medical Clinic C, 67063 Ludwigshafen, Germany; ^3^Department of Rheumatology, Osteology and Physical Therapy, William G. Kerckhoff Foundation, Justus Liebig University Giessen, 35385 Giessen, Germany

## Abstract

*Background*. Previous research on the combined association of 25-hydroxyvitamin D [25(OH)D] and exocrine pancreas insufficiency may have been limited by restricted age variability and a lack of representation of both body weight and body mass index. There are still too few conclusive reports about conspicuous vitamin D metabolism according to pancreatic fecal elastase 1 (FE1) in obese patients. *Methods*. Between May 2004 and July 2008, we investigated in 125 female patients with obesity syndrome at an average age of approximately 52.9 years as well as in age-matched 80 healthy female controls the prevalence of pancreas insufficiency. Serum levels of PTH, total calcium, and D_3_ vitamins calcitriol and calcifediol, as well as the concentration of fecal elastase 1 (FE1) were determined in patients and controls. *Results*. In 75 female nondiabetic patients with obesity syndrome (BMI 35 ≤ 40 kg/m^2^), calcifediol was markedly decreased (25.0 ± 4.9 ng/mL) compared to controls (50.2 ± 14.7 nmol/L; *P* < 0.01). FE1 level was significantly decreased in obese subjects compared to controls (* P* < 0.01). Calcifediol was significantly lower in patients with morbid obesity (for calcifediol, * P* < 0.05). *Conclusion*. In obese females, pancreatic FE1 in feces confirms the extent of vitamin D supply, and thus shows a vitamin D_3_ deficiency, depending on the loss of stool content. There seems to be a connection between the loss of exocrine function and the increasing body mass index. Pancreas insufficiency, as detected by low FE1 concentrations, is frequent in obese patients. However, the BMI is an additional factor for lowered fecal excretion of FE1.

## 1. Introduction

Vitamin D deficiency, as reflected by circulating 25-hydroxyvitamin D (25[OH]D) levels less than 20 ng/mL, is prevalent in as many as one-half of middle-aged to elderly adults in developed countries [1]. Additionally, vitamin D status is strongly associated with variation in subcutaneous and especially visceral adiposity [2]. Female patients without any history of diabetes or pancreatitis but with deficiency of fecal elastase 1 show alterations of the vitamin D metabolism. These resulted from the impaired exocrine pancreas function as compared to indirect test method: estimation of fecal elastase [3].

Deficiency of elastase 1 in feces (FE1) has previously been regarded to be revealed sensitivities of 14%, 87%, and 95% for the Cambridge grades I, II, and III, and correlated significantly with this classification of severity of chronic pancreatitis [4]. FE1 is, compared to the “gold standard,” the secretin caerulein test, a highly sensitive and specific tubeless pancreatic function test [5]. In the diagnosis of chronic pancreatitis there is a parallelism between exocrine function and ERCP results [6], even using FE1 for description of pancreatic insufficiency [7, 8]. One field which has until now received little attention is the changes of vitamin D_3_ serum levels subjected to the different severity grades of pancreatic exocrine insufficiency. The consequences of exocrine insufficiency might be relevant for serum levels of lipid soluble vitamin D_3_. However, conspicuous vitamin D deficiency in patients with chronic pancreatitis has only been described by very few authors until now [9–13]. Accordingly, one of the most obvious causes of elevated PTH serum levels, impaired and lowered vitamin D_3_, exocrine insufficiency with consecutive malassimilation and resulting insufficient vitamin D-supply should be further investigated.

Exocrine insufficiency of pancreas is frequent in both type 1 and type 2 diabetes mellitus [14]. The connection between obesity syndrome and the manifestation of diabetes mellitus is well accepted. Overweight or obese subjects without metabolic syndrome were at increased risk for diabetes [15].

Although the lowered FE1 has been investigated in subjects with both diabetes mellitus and hypovitaminosis D, fecal pancreas elastase 1 in subjects with obesity syndrome and vitamin D deficiency has not been demonstrated. Thus the aim of the present study was undertaken to determine the impact of body weight and nutritional status on FE1 and 25-hydroxyvitamin D (25[OH]D) serum levels.

## 2. Material and Methods

### 2.1. Patients

Between May 2004 and July 2008, 125 female outpatients (aged from 52.9 ± 10.51 years) with obesity syndrome were included in our studies. They were admitted to hospital for work-up extreme weight gain, abnormalities of body composition, obesity, or infertility syndrome. Exclusion criteria were male sex, age under 35 or over 65 years, steatorrhea, pancreatic-biliary obstructions, actual and relevant alcohol consumption, medication with influence on osteological and/or endocrine parameters (heparin, ketoconazol, glucocorticoids, thiacide-diuretics, psychopharmacological agents, and carbamazepin), altered kidney function, pregnancy, and chronic or severe concomitant diseases. Based on the assumption of an interaction between exocrine and endocrine pancreatic function we excluded patients with a diabetes mellitus [15]. The selected patients were studied in detail with history and physical examination, including detailed neurological examination. Body mass index (BMI) was calculated from the height and weight measurements of the patients. To examine adiposity and body size separately, we repeated multivariable analyses of adiposity measures within subgroups defined by BMI category (<25, 30 to <35, 35 to <40, and ≥40 kg/m^2^). 

 Data regarding total vitamin D intake from supplements and diet were obtained using a detailed food-frequency questionnaire. 80 healthy female subjects between 39 and 60 years of age with a BMI ≤ 25 kg/m^2^ served as controls [3].

### 2.2. Biochemical Measurements

Blood samples were taken from all participants every morning at the same fixed time. We did not consider the specific circannual periodic of the vitamin D metabolism. The specific serum parameters of this study were PTH (“intact PTH 1–84 IRMA Kit” from Nichols Institute Diagnostics, San Juan Capistrano, California, USA), calcitriol (“1,25 (OH)_2_ Vitamin D”-kit from Immun Diagnostik, Bensheim, Germany; competitive radio receptor assay), and calcifediol (“25 (OH)_2_ Vitamin D” kit from Immun Diagnostik, Bensheim, Germany; competitive protein binding assay). Pancreatic elastase 1 (“Pankreatic Elastase 1”-kit from ScheBo Biotech, Giessen, Germany; double-sided enzyme immuno-assay) was determined in the feces of all participants.

### 2.3. Statistical Analysis

Results are presented by mean values and standard deviation. The following methods were applied for statistical analysis: a single factor variance analysis, the Scheffé-Test, the nonparametric Kurskal-Wallis-Test with subsequent Dunn-Test, as well as the *t*-test for independent random samples with and without the Welche's correction. Pearson's correlation coefficient and also the non-parametric Spearman correlation coefficient were applied for investigating any connections [16, 17].

## 3. Results

### 3.1. Prevalence of Vitamin D Deficiency

A total of 125 obese female patients and 80 controls were included in the study. The 25 (OH)_2_ vitamin D deficiency (<20 ng/mL) was more frequent among individuals with high BMI (30 to <35, 35 to <40 and ≥40 kg/m^2^) compared with those with low BMI; (<25 57.7% versus 9.7%, *P* < 0.01). 

Furthermore, the vitamin D metabolism did not differ significantly within the various subgroups: BMI 30 < 35 kg/m^2^ (*N* = 25); 35 < 40 kg/m^2^ (*N* = 75) > 40 kg/m^2^ (*N* = 25) as well as extremely decreased compared to the controls (*P* < 0.01; Tables 1 and 3): calcitriol in patients with obesity was slightly decreased, but did not differ significantly within the various BMI groups (*P* > 0.05). Calcifediol was not significantly different within the various BMI groups (*P* = 0.07). Nevertheless, both D_3_-vitamins were lower in female obese patients.

### 3.2. Prevalence of Fecal Pancreas Elastase 1 Deficiency

The majority of women with obesity syndrome presented a lowered fecal excretion of pancreatic elastase 1. These observations were aggravated by an elevated BMI of the subjects (Table 1,  *P* < 0.01, Table 2). Additionally, FE1 in obese patients correlated significantly with both D_3_-vitamins (*P* < 0.01; Table 3). 

Concerning estimation of PTH levels in serum, there was no correlation both between the fecal elastase 1 and the D_3_-vitamins calcitriol and calcifediol. In 125 female obese patients only three patients (48, 51, 53 years) showed milder cases of newly diagnosed primary hyperparathyroidism. At diagnosis of primary hyperparathyroidism events involving gastric disorders, for example, gastric ulcers, were prevalent in history of both patients. However, there was no evidence of an acute pancreatitis in case history or abdominal ultrasound examination.

## 4. Discussion

We found an increased prevalence of lowered fecal excretion of pancreatic elastase 1 (FE1) in female obese patients. This alteration of the fecal excretion of FE1 is independent on disturbances of glucose metabolism, disorders of pancreas, or gall bladder. 

Data about exocrine insufficiency of pancreas in obesity syndrome are rare. Concerning the body mass index, Hahn et al. [18] demonstrated the loss of an influence on exocrine pancreas function by using a secretion caerulein test in diabetic subjects with different BMI. Using the FE1 for estimation of exocrine pancreas function, diabetic individuals with excess weight (BMI > 25) may be at increased risk for underlying exocrine pancreatic insufficiency. In diabetes mellitus (*n* = 42), 11 patients with excess weight presented a FE1 < 200 microg/g, whereas four patients with a BMI < 25 presented this result (*P* < 0.05) [19]. On the other hand, the excess weight does not seem to affect the pancreas exocrine function when it is not associated with diabetes mellitus [19]. 

To our knowledge, this is the first study dealing with the link between FE1 and the respective serum levels of vitamin D_3_ in obese subjects. Assuming that FE1 in feces is the representative marker for an exocrine insufficiency during a chronic pancreatitis, Dutta et al. [9] already described a frequent loss of lipid soluble vitamins, even vitamin D, in patients with chronic pancreatitis and exocrine insufficiency. Accordingly, the present study showed a highly significant correlation between FE1 and vitamin D_3_, which was more pronounced for calcifediol than calcitriol. Under the aspect of an exocrine pancreas insufficiency with resulting malabsorption for vitamin D in patients with chronic pancreatitis, FE1 gains significance hereby as a pancreas function test. Therefore, vitamin D_3_ deficiency would be very dependent upon the severity grade of exocrine insufficiency, represented here by the FE1 in obese patients. Assuming that steatorrhea is the obvious symptom of exocrine insufficiency in patients with chronic pancreatitis, as reported by Twersky and Bank [20], that its existence as well as occurrence is dependent upon the severity of exocrine dysfunction and fat supply in food, the results from Dutta et al. [9], Dibble et al. [10], and also Nakamura et al. [11] contradict the above-mentioned statement. Dutta et al. [9] could not find any connection between measured lipid soluble vitamins (including calcifediol) and severity of steatorrhea in patients with chronic pancreatitis. Likewise, Dibble et al. [10] obtained the same results by applying calcifediol only, and Nakamura et al. [11] by applying all lipid soluble vitamins (including calcitriol and calcifediol), except vitamin E, in all patients. Also, Haaber et al. [13] described the lack of difference for calcitriol and calcifediol depending on the exocrine insufficiency as well as on the duration of the disease in patients with chronic pancreatitis. Nevertheless, all these observations are not suitable to invalidate the link of elastase 1 values in feces, severity of disease, and vitamin D deficiency in patients with exocrine insufficiency. According to Twersky and Bank [20] and DiMagno et al. [21], the parameter of steatorrhea is too variable for a precise description of exocrine function of the pancreas, and therefore, statements by Dutta et al. [9], Dibble et al. [10], and Nakamura et al. [11] are in this sense less representative. The results from Haaber et al. [13] also lose their significance, because enzymes were substituted in patients with exocrine pancreatic insufficiency. On the basis that under normal circumstances 80–90% of experimentally applied, radioactively labelled vitamin D_3_ is absorbed by the intestines, although only 40% in patients with pancreatic insufficiency [22], exocrine pancreatic function gains significance and supports our own results with corresponding evaluation of FE1. Furthermore, it is conceivable that FE1 plays an independent role with regard to vitamin D_3_ supply in the organism. Since by passing the intestines, FE1 changes to a complete protein sterol complexity by loading neutral steroids [23], and since vitamin D_3_ is also a sterol molecule, there is a hypothetical cross-link here. Therefore, there are still queries regarding the importance of sterol linking in elastase 1 in interaction with its excretion status for vitamin D_3_ supply. 

As vitamin D deficiency and hyperparathyroidism are relatively common, a coexistence of these conditions must be considered. Vitamin D deficiency may increase the severity of primary hyperparathyroidism (presence of larger adenomas, higher PTH levels, and greater bone turnover). Nevertheless, some of the biochemical features of primary hyperparathyroidism can be masked by the coexisting vitamin D deficiency leading to an inappropriate therapeutic management of these patients [24]. Although several lines of evidence indicate that serum PTH may be associated with metabolic disturbances, to our knowledge, only few studies have addressed the combined effect of serum PTH and vitamin D on metabolic syndrome and visceral adiposities. These reports, however, diverge in their conclusions. The finding of a close relationship between metabolic syndrome and PTH level is in contrast with the negative results of a previous study of severely obese subjects [25, 26], but extend the results from a study of older mainly nonobese men to be valid in adult Caucasian treatment seeking morbidly obese women and men. Further, although Hjelmesæth et al. found a significant inverse correlation between 25(OH)D and PTH, the group could not confirm any association between 25(OH)D and metabolic syndrome as shown by others [25, 27]. 

Our study also had limitations. First, the cross-sectional design makes it difficult to establish a cause-effect relationship. Second, our results may not be valid in diagnosing diabetes mellitus by using an oral glucose tolerance test. The exclusion criteria of a pancreatitis were performed only by ultrasound examination. We did use a magnetic resonance imaging for the description of the pancreas morphology. Finally, we cannot exclude the possibility that referral of patients to a tertiary care centre might have introduced a selection bias.

In summary, obese female patients present a lowered excretion of FE1 and show alterations of the vitamin D metabolism. These resulted from the impaired exocrine pancreas function as compared to indirect test method: estimation of FE1. The estimation of FE1 is a well-founded indirect test for determination of vitamin D supply in obese female subjects as well.

## Figures and Tables

**Table 1:** Age, faecal elastase 1, calcitriol, and calcifediol (means ± standard deviation) in female patients with obesity syndrome and controls. *P* < 0.05 indicates a significant difference between the patient-collectives allotted by the body mass index (BMI).

Parameters	Controls (*N* = 80)	BMI** [**kg/m^2^]			*P*
		I 30 ≤ 35 (*N* = 25)	II 35 ≤ 40 (*N* = 75)	III ≥40 (*N* = 25)	
Age (years)	54.8 ± 12,1	51.6 ± 9,3	53.5 ± 7.0	52.4 ± 8.6	n.s.
Faecal elastase 1 (*μ*g/g)	665.9 ± 117.4	414.7 ± 71.9	299.6 ± 44.8	224.1 ± 69.5	*P* < 0.01
Calcitriol (pg/mL)	41.0 ± 8.3	41.7 ± 8.2	34.4 ± 5.1	30.5 ± 6.1	n.s.
Calcifediol (ngl/mL)	50.2 ± 14.7	34.1 ± 12.5	25.0 ± 4.9	18.8 ± 7.3	*P* < 0.01
PTH (pg/mL)	41.3 ± 10.2	44.8 ± 21.3	51.4 ± 15.7	54.1 ± 20.9	n.s.

**Table 2:** Comparison of serum PTH, calcitriol and calcifediol, and fecal elastase 1 content, between different patient-collectives allotted by the body mass index (BMI) and controls. *P* < 0.05 indicates a significant difference between patients with BMI and controls.

Parameters	Comparison of error probabilities of variation between patients and controls (*N* = 80) concerning BMI (kg/m^2^)		
	Controls (*N* = 80)	30 ≤ 35 (*N* = 25)	>35 (*N* = 100)
Faecal elastase 1 (*μ*g/g)	*P* < 0.01	*P* < 0.01	*P* < 0.01
Calcitriol	*P* < 0.01	n.s.	*P* < 0.01
Calcifediol	*P* < 0.01	*P* = 0.05	*P* < 0.01
PTH	n.s.	n.s.	n.s.

**Table 3:** Correlation between vitamin D_3_ and elastase 1 in feces in obese female patients. *P* < 0.05 indicates a significant correlation.

*N* = 125	Vitamin D_3_	
Parameter	Calcitriol	Calcifediol
*Elastase 1 in feces*		
Correlation Pearson	0.721	0.658
*P*	*P* < 0.05	*P* < 0.01
